# Successful rescue of aconitine poisoning with extended cardiopulmonary resuscitation combined with hemoperfusion: A case report and literature review

**DOI:** 10.1097/MD.0000000000047787

**Published:** 2026-02-20

**Authors:** Hongyu Chen, Xiaoping Huang, Xuelan Wen, Shaochun Lu, Zhihong Zhang, Gang Yuan

**Affiliations:** a Emergency Department, The Affiliated Traditional Chinese Medicine Hospital, Southwest Medical University, Luzhou, China; b Electrocardiogram Room, The Affiliated Traditional Chinese Medicine Hospital, Southwest Medical University, Luzhou, China; c Department of Intervention and Vascular, The Affiliated Traditional Chinese Medicine Hospital, Southwest Medical University, Luzhou, China.

**Keywords:** aconitine poisoning, case report, electrical storm, extended cardiopulmonary resuscitation, hemoperfusion

## Abstract

**Rationale:**

Aconitine, a highly toxic alkaloid derived from *Aconitum* plants, exhibits a narrow therapeutic window, with a poisonous dose as low as 0.2 mg and lethal dose of 2 to 5 mg. Currently, no specific antidote exists for aconitine poisoning, which frequently triggers refractory electrical storms and cardiopulmonary arrest, culminating in high mortality. Elderly patients face particularly dire prognoses due to diminished physiological reserves. This study evaluated the efficacy of extended cardiopulmonary resuscitation (CPR) combined with hemoperfusion (HP) as a rescue strategy for aconitine-induced cardiac arrest.

**Patient concerns:**

A 90-year-old female presented to the emergency department with a 2-hour history of dizziness, palpitations, and generalized numbness, followed by impaired consciousness after accidental ingestion of aconitine-containing medicinal wine. On admission, the patient exhibited respiratory failure, circulatory collapse, and malignant arrhythmias, including sustained ventricular tachycardia and fibrillation, which rapidly progressed to cardiac arrest.

**Diagnoses:**

The patient was diagnosed with acute severe aconitine poisoning complicated by cardiopulmonary arrest based on a history of exposure, characteristic clinical manifestations, and electrocardiographic findings.

**Interventions:**

A multidisciplinary rescue protocol was immediately implemented: endotracheal intubation and mechanical ventilation were performed, followed by continuous high-quality CPR using a Lund University Cardiopulmonary Assist System mechanical resuscitation device for a cumulative duration exceeding 200 minutes. Once CPR has established effective circulatory support, HP therapy should be initiated immediately to remove toxins from patient circulation. Combination of pharmacotherapy with antiarrhythmic agents (amiodarone and lidocaine) and vasoactive support (norepinephrine) to stabilize rhythm and perfusion.

**Outcomes:**

After prolonged resuscitation and multimodal detoxification, the patient’s electrical storm resolved, with restoration of sinus rhythm. She regained consciousness on hospital day 3, was successfully extubated on day 4, and was discharged after 16 days without significant neurological deficits. A 3-month follow-up confirmed sustained recovery of cardiopulmonary and cerebral function.

**Lessons:**

This case demonstrates that extended high-quality CPR combined with HP is a feasible and effective intervention for aconitine poisoning–related cardiac arrest, particularly when conventional antiarrhythmic and electrical therapies fail. Success depends on early recognition, persistent resuscitation, and integrated toxin removal strategies.

## 1. Introduction

Aconitine, a highly toxic diester-diterpenoid alkaloid derived from plants of the genus *Aconitum* (Ranunculaceae family), has an extremely narrow therapeutic window, with a toxic dose (approximately 0.2 mg) dangerously close to the lethal dose (2–5 mg).^[1]^ Its toxicity mechanism is complex and rapid, primarily involving the sustained activation of voltage-gated sodium channels, dysregulation of the autonomic nervous system, and disruption of myocardial calcium homeostasis, collectively leading to multisystem damage.^[2]^ Following oral ingestion, the latent period was short (ranging from minutes to 2 hours). Poisoned individuals typically present with nausea, vomiting, paresthesia, and palpitations, whereas severe cases can rapidly progress to generalized numbness, coma, refractory electrical storms (ES), or even cardiac arrest.^[2,3]^

Clinically, aconitine poisoning is more prevalent in middle-aged and elderly populations, particularly among those who self-administer *Aconitum*-based preparations (such as *chuanwu*, *caowu*, or *fuzi*) externally or orally for rheumatism-related conditions.^[4,5]^ Its incidence exhibits distinct geographic and seasonal patterns, with heightened risks observed in regions of China where wintertime tonic-consumption customs are practiced.^[5]^ Improper processing, overdosing, accidental ingestion of aconitine-containing preparations, and intentional poisoning or suicide are also documented causes.^[6–9]^ Currently, no specific antidote exists for aconitine poisoning, making early recognition and comprehensive supportive care central to its management.^[2,3]^ Treatment strategies should be systematic and stratified as follows.

### 1.1. Gastrointestinal decontamination

For patients presenting within 1 hour of ingestion, cautious measures (e.g., induced emesis or gastric lavage) may be considered with attention to aspiration risk.^[3]^

### 1.2. Life support and arrhythmia control

Endotracheal intubation with mechanical ventilation, antiarrhythmic drugs (e.g., amiodarone and flecainide), electrical cardioversion/defibrillation, high-quality cardiopulmonary resuscitation (CPR), and vasoactive agents.^[10]^

### 1.3. Toxin removal techniques

Hemoperfusion (HP) effectively adsorbs toxins with molecular weights ranging from 500 to 5000 Da, making it particularly suitable for aconitine (molecular weight 645.7 Da), which exhibits a high protein-binding capacity.^[11]^

### 1.4. Advanced life support

In cases of refractory cardiac arrest that are unresponsive to conventional therapy, extended CPR should be implemented. Venoarterial extracorporeal membrane oxygenation (VA-ECMO) can provide circulatory and oxygenation support, although its use is often limited by the availability of healthcare resources.^[5,12]^

Therefore, timely implementation of the integrated management strategies outlined above is critical for the successful resuscitation of critically ill patients. This report describes a case of a 90-year-old female who developed ES and cardiac arrest after accidental ingestion of aconitine-containing medicinal wine. The patient was successfully rescued through extended CPR (>200 minutes) combined with intermittent HP. In conjunction with a literature review, this article systematically elaborates on the pathophysiology, stratified management, and optimization of intervention strategies for aconitine poisoning, with the aim of providing a reference for standardized clinical management.

## 2. Case presentation

A 90-year-old female patient was admitted to the emergency department due to “dizziness and palpitations for 2 hours and impaired consciousness for 1 hour after oral intake of medicinal wine.” Medical history revealed that before 2 hours, she had accidentally ingested medicinal wine infused with aconite and rapidly developing palpitations, dizziness, and limb numbness. Emesis induced outside the hospital did not alleviate the symptoms, which gradually worsened and progressed to chest tightness, palpitations, dyspnea, and generalized numbness. One hour later, she developed confusion, accompanied by paroxysmal convulsions and incontinence. The patient had suffered from rheumatic pain for decades and had long used aconitine-containing medicinal wine for topical application with fair results and no other significant medical history.

**Figure 1.**
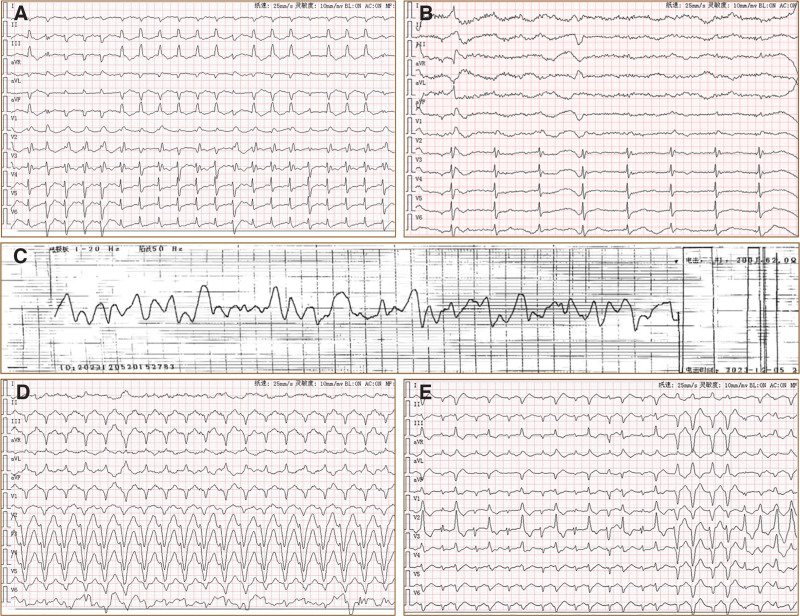
The ECG during the rescue process. (A) Ectopic rhythm, sustained VT, partially manifesting as bidirectional VT; (B) ectopic rhythm, accelerated idioventricular rhythm; (C) ectopic rhythm, VF (ECG waveform recorded during electrical defibrillation); (D) ectopic rhythm, sustained VT; and (E) ectopic rhythm, sustained VT, partially manifesting as bidirectional VT. ECG = electrocardiogram, VF = ventricular fibrillation, VT = ventricular tachycardia.

On admission, the patient was restless, with weak breathing and cyanosis of the lips and nail beds. Cardiac auscultation revealed a weak heart rate. She was transferred to the resuscitation room at 17:05. Cardiac monitoring showed heart rate = 200 bpm, respiratory rate = 15 bpm, blood pressure = 101/56 mm Hg, peripheral oxygen saturation = 68%, and Glasgow Coma Scale score = 8 (E2M4V2). An arterial blood gas analysis revealed decompensated metabolic acidosis. Based on the medical history and auxiliary examinations, the patient was diagnosed with acute severe aconitine poisoning complicated by persistent ventricular tachycardia (VT). We immediately established multiple intravenous access paths and performed an endotracheal intubation for mechanical ventilation to provide life support. Simultaneously, an emergency multidisciplinary consultation (cardiology, pulmonology, and clinical pharmacy) was initiated to formulate rescue measures jointly. An electrocardiogram (ECG) at 17:15 showed ectopic rhythm and sustained VT, partially manifesting as bidirectional VT (Fig. 1).

**Figure 2.**
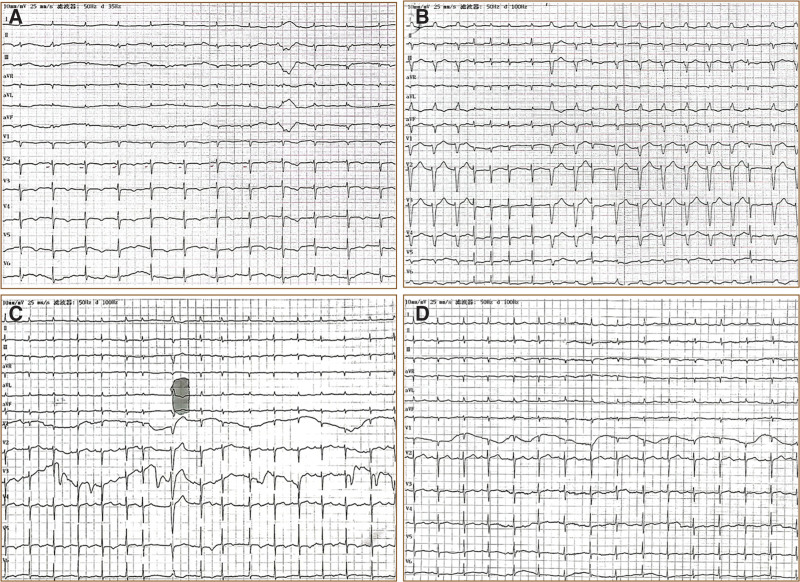
The ECG after rescue. (A) Sinus rhythm, low limb lead voltage, prolonged ST segment, T wave changes, prolonged (Q–T) interval; (B) sinus tachycardia with irregularity, frequent ventricular premature contractions (some in pairs), nonsustained VT with incomplete atrioventricular interference dissociation, ST-T changes; (C) sinus rhythm, occasional atrial premature contractions, occasional ventricular premature contractions, ST-T changes; and (D) sinus rhythm, T wave changes. ECG = electrocardiogram, ST-T = ST segment and T wave, VT = ventricular tachycardia.

The diagnosis of aconitine poisoning in the patient was definitive. Given this critical condition, we implemented multiple rescue measures, and the key procedures are summarized in Table 1. Dynamic changes in the ECG results of the patient are shown in Figures 1 and 2. Following prolonged CPR and multiple sessions of intermittent hemoperfusion, the patient’s vital signs gradually stabilized. Consciousness regained on the third day after admission, and successful extubation was achieved on the fourth day. With subsequent active symptomatic treatment, the patient’s condition gradually improved. Follow-up ECG revealed sinus rhythm (Fig. 2). The patient was discharged 16 days after the hospitalization. Although her memory was slightly impaired at discharge, she exhibited no other neurological deficits or cardiopulmonary dysfunctions. At the 3-month follow-up, her general condition remained favorable, with significant memory improvement compared with the time of discharge.

**Table 1:** The main rescue measures and efficacy of the patient.

Date	Time line	Patient status	Treatment
12.05	17:05	Respiratory and circulatory failure, GCS 8 (E2M4V2).	Tracheal intubation and mechanical ventilation.
	17:10	VT, HR 200 bpm.	Amiodarone 0.15 g IV, and 1 mg/min was maintained by intravenous pumping.
	17:15	VT, HR 120–130 bpm (Fig. 1A).	Lidocaine injection 100 mg IV.
	17:20	BP decreased to 82/43 mm Hg.	Electrical cardioversion.
	17:25–18:00	VF and loss of carotid pulse (Fig. 1C).	Initiate CPR with the mechanical resuscitation device LUCAS, compression depth 5 cm, compression rate 105 compressions per minute; defibrillation; epinephrine pushes every 5 minutes; and simultaneously give magnesium sulfate, atropine, magnesium-containing polarizing fluid, and 5% sodium bicarbonate injection intravenously.
	18:00–19:20	The spontaneous HR is restored in short bursts but cannot be maintained, with repeated VF and pulseless VT.	Continue CPR, multiple defibrillations; invasive arterial BP monitoring; mild hypothermia treatment.
	19:30	Invasive BP 60 mm Hg.	Continue infused norepinephrine to maintain a mean arterial pressure above 65 mm Hg and initiate the first HP with an extracorporeal blood flow rate of 80–100 mL/min.
	20:52	Accelerated ventricular rhythm (Fig. 1B).	Respiratory and circulatory support, continuous HP.
	21:32	Spontaneous rhythm with sustained palpable carotid pulse.	Stop chest compressions.
	22:00	HR 64 bmp, R 20 bmp, BP 93/54 mm Hg (norepinephrine maintenance), SPO_2_ 95%, GCS 2T.	The first HP was completed.
	22:22	VT (Fig. 1D).	Continuous respiratory and circulatory support to stabilize the internal environment.
12.06	02:00–04:00	VT, BP, and SPO_2_ were stable.	Second HP with an extracorporeal blood flow rate of 150–180 mL/min.
	05:25	Bidirectional VT (Fig. 1E).	Continuous respiratory and circulatory support.
	11:00–13:00	VT, BP, and SPO_2_ were stable.	The third HP with an extracorporeal blood flow rate of 180–200 mL/min.
	20:55	Sinus rhythm (Fig. 2A).	Discontinue vasopressors and antiarrhythmic drugs.
12.07		Sinus tachycardia with irregular rhythm (Fig. 2B), GCS 6T (E2M4).	Respiratory support, try to wean off the ventilator.
12.08		Vital signs were stable, with sinus rhythm (Fig. 2C), and GCS 10T.	The patient was successfully weaned from the ventilator and extubated.

BP = blood pressure, CPR = cardiopulmonary resuscitation, E = eye opening, GCS = Glasgow Coma Scale, HP = hemoperfusion, HR = heart rate, LUCAS = Lund University Cardiopulmonary Assistance System, M = motor response, R = respiratory rate, SPO_2_ = peripheral oxygen saturation, T = tracheostomy, V = verbal response, VF = ventricular fibrillation, VT = ventricular tachycardia.

## 3. Discussion

Aconitine alkaloids are widely distributed in all parts of *Aconitum* plants, with a toxic dose of approximately 0.2 mg and lethal dose of 2 to 5 mg.^[1]^ After oral ingestion, aconitine is rapidly absorbed, characterized by a short latency period and rapid onset of toxicity, typically manifesting as symptoms within minutes to 2 hours.^[2,3]^ The mechanisms of aconitine poisoning are complex, and current research has confirmed that it primarily includes the following: Modulation of voltage-gated sodium channels: aconitine has a strong affinity for binding site 2 of the voltage-gated sodium channel α-subunit. Activation of this channel leads to sustained sodium influx, increases in intracellular sodium concentration, and causes persistent depolarization, thereby triggering arrhythmias.^[2]^ Excitation of the vagus nerve: aconitine suppresses the automaticity of the sinoatrial and atrioventricular nodes while stimulating ectopic pacemakers, resulting in various arrhythmias,^[2]^ including tachyarrhythmias, bradyarrhythmias, and even cardiac arrest. Induction of a refractory period due to prolonged channel activation: as the ion channels remain open, myocardial cells enter a refractory state for further stimulation. After the initial hyperexcitability phase, patients may enter a suppression phase resembling sodium channel blockade, potentially leading to life-threatening ventricular arrhythmias.^[13]^ Modulation of calcium channels: aconitine interferes with cellular metabolic pathways and reduces acetylcholine release, exacerbating arrhythmias.^[10]^ The inhospital mortality rate for aconitine poisoning is 15%.^[2]^ Once aconitine poisoning accompanied by severe arrhythmias is diagnosed, an emergency intervention is required.

Currently, there is no specific antidote for aconitine poisoning, emphasizing the importance of early intervention and symptomatic supportive care as the mainstay of treatment. Studies^[3]^ suggest that, if medical attention is sought within 1 hour of ingestion, gastrointestinal decontamination measures (including induced vomiting, gastric lavage, catharsis, and enema) may be attempted. However, caution is warranted because of the rapid absorption of aconitine, which can lead to rapid onset of toxic symptoms. Symptomatic treatment focuses on maintaining airway patency, ensuring adequate ventilation and oxygenation, and supporting cardiac electrophysiology and the necessary circulatory function. Key methods include endotracheal intubation and mechanical ventilation, antiarrhythmic drugs, electrical cardioversion/defibrillation, vasoactive medications, and ECMO. Additionally, enhancing the elimination of absorbed aconitine is crucial using common methods, including forced diuresis and HP. The patient had a clear history of aconitine exposure, classic clinical manifestations of poisoning, and characteristic ECG findings, which confirmed the diagnosis of acute severe aconitine poisoning. However, she was admitted 2 hours after ingestion, missing a window for gastrointestinal decontamination. On admission, the patient presented with respiratory and circulatory failure and malignant arrhythmias. Thus, immediate priorities focused on protecting the airway and stabilizing respiration and circulation as primary goals.

ES was defined as 3 or more episodes of sustained ventricular arrhythmia (including appropriate implantable cardioverter-defibrillator shocks) within 24 hours, with each episode separated by at least 5 minutes. Malignant arrhythmias are the leading cause of death owing to aconitine poisoning.^[3]^ To date, no single antiarrhythmic drug has demonstrated specific efficacy against aconitine-induced arrhythmias. Reports^[12]^ show that flecainide, mexiletine, procainamide, amiodarone, lidocaine, and magnesium sulfate have been used in cases of aconitine-induced cardiotoxicity. Among these, flecainide and amiodarone have shown the strongest associations with sinus rhythm restoration in a limited number of human case studies. Lidocaine (Class Ib antiarrhythmic) blocks myocardial sodium channels, reducing Na^+^ influx and cellular automaticity, thereby suppressing ventricular ectopy. However, it appears to be minimally effective against aconitine-induced ventricular arrhythmias, as it achieves sinus rhythm restoration in only 1 of over 30 instances.^[3,10]^ The European Resuscitation Council Advanced Life Support Guidelines recommend electrical cardioversion as the primary treatment for cardiogenic shock or cardiac arrest caused by ES. However, due to the unique arrhythmogenic mechanism of aconitine, electrical cardioversion often yields suboptimal results. Despite treatment with amiodarone, lidocaine, and electrical cardioversion, the patient’s arrhythmias remained refractory.

Due to reduced physiological reserves in multiple organ systems, elderly patients exhibit a diminished capacity to maintain homeostasis when facing disease or injury. This leads to significantly lower CPR success rates, with an overall survival rate of ≤11.1%.^[14]^ Treatment of aconitine poisoning in elderly patients is even more challenging. Therefore, the early recognition of cardiac arrest and prompt initiation of CPR are crucial. The 90-year-old patient had poor baseline health. Following the 2020 American Heart Association CPR guidelines, we implemented the following under intensive monitoring: early CPR initiation upon detecting malignant arrhythmia-induced cardiogenic shock and cardiopulmonary arrest and mechanical CPR with a Lund University Cardiopulmonary Assist System after securing the airway (via prior intubation), ensuring consistent, high-quality chest compressions during prolonged resuscitation. Prolonged CPR duration is recognized as a key intervention for aconitine-induced ES and cardiac arrest.^[3]^ This patient underwent a cumulative resuscitation time exceeding 200 minutes, during which she experienced multiple episodes of VT, pulseless VT, and ventricular fibrillation. The application of the Lund University Cardiopulmonary Assist System device during resuscitation ensured the stability and continuity of chest compressions during this prolonged effort,^[15]^ guaranteeing effective perfusion of vital organs and creating conditions for toxin clearance via HP.

HP removes solutes through adsorption, demonstrating high efficacy for middle-molecular-weight substances (500–5000 Da), with small volume distribution and high protein-binding capacity.^[11]^ This technique establishes an artificial dual-access blood-purification circuit. A blood pump draws blood that is extracorporeally rich in harmful solutes. As blood flows through an adsorption column, harmful solutes are separated using adsorption principles. The purified blood is subsequently returned to the systemic circulation, accomplishing toxin clearance through cyclic processing. An ideal adsorbent material should possess a high adsorption capacity and selectivity, good biocompatibility, stable physicochemical properties, and rapid adsorption kinetics. Carbon-based materials, resins, and novel composite materials have improved the safety and efficacy of HP in clinical applications through advanced coating technologies, thereby reducing adverse reactions.^[16]^ Aconitine is highly lipophilic with a relative molecular mass of 645.7. The half-life of aconitine is long and varies significantly, with a maximum duration of up to 17.8 hours, and it can differ between individuals and is difficult to predict.^[3]^ When bound to proteins in the blood, they can form complexes with higher molecular weights. Research^[11]^ has shown that HP can effectively reduce serum aconitine concentrations, shorten the duration of its toxic effects, effectively reduce the risk factors for ES, and play a significant role in terminating ES. HP also reduces the metabolic burden on organs, such as the liver and kidneys, and lowers the incidence of multiple organ dysfunction syndrome. In this patient, timely HP to clear toxins played a crucial role in ending ES when drug therapy, cardioversion, and defibrillation failed to terminate the ES. However, because of the high protein-binding rate, prolonged half-life, redistribution of toxins, and saturation of adsorption materials, multiple sessions of HP are required to effectively reduce the blood concentration.^[17]^ Furthermore, the circulating blood volume is insufficient during CPR. Therefore, we administered norepinephrine to maintain an effective mean arterial pressure for vital organ perfusion while implementing a low-flow HP strategy. Ultimately, comprehensive cardiopulmonary-cerebral resuscitation was achieved, and the patient was successfully rescued.

In recent years, VA-ECMO has gained increasing attention for the treatment of refractory cardiac arrest due to poisoning.^[12]^ VA-ECMO provides temporary circulatory and oxygenation support for patients with cardiac arrest and mitigates ischemia-reperfusion injuries. Thus, in cases of aconitine-induced cardiac arrest that are unresponsive to pharmacotherapy or electrical cardioversion, VA-ECMO serves as a critical therapeutic intervention.^[4]^ However, the high cost of ECMO systems has limited their widespread adoption in many medical centers. In settings where ECMO is unavailable, prolonged CPR combined with HP remains a clinically vital approach for managing refractory cardiac arrest caused by aconitine poisoning.

## 4. Limitations

Despite the ultimate therapeutic success of this case, the present study has several limitations. First, constrained by local medical resources, the implementation of certain therapeutic techniques that might have been more ideally suited to this case, such as ECMO, was not feasible. Second, dynamic monitoring of serum aconitine concentrations was not performed during the treatment course, precluding quantitative assessment of the toxin clearance efficiency of HP. Furthermore, clinical reports on the synchronized implementation of CPR and HP remain relatively scarce. Critical parameters for this combined therapeutic modality, such as the timing of perfusion initiation and optimal blood flow settings, lack standardized guidelines. Consequently, the safety and efficacy of this approach require further validation through additional studies.

## 5. Conclusions

The management of severe aconitine poisoning requires the systematic implementation of a tripartite strategy encompassing “detoxification, perfusion maintenance, and rhythm stabilization.” During the emergency phase, the primary objective is the rapid control of malignant arrhythmias, with timely toxin removal serving as a critical measure to terminate arrhythmias. In the event of cardiac arrest, high-quality, sustained CPR must be rigorously maintained. Adherence to evidence-based medical principles, coupled with a persistent and unwavering commitment to treatment, constitutes the cornerstone of successful patient survival.

## Acknowledgments

This study was funded by the Sichuan Provincial Science and Central Guidance Local Exploration Fund Project (2024ZYD0103). We thank Mr. Xinglin Chen (Medical Records Department of The Affiliated Hospital of Southwest Medical University) for providing the medical records and relevant materials.

## Author contributions

**Data curation:** Hongyu Chen, Xiaoping Huang, Xuelan Wen, Shaochun Lu.

**Investigation:** Hongyu Chen, Xiaoping Huang, Xuelan Wen, Shaochun Lu.

**Resources:** Hongyu Chen, Xiaoping Huang, Xuelan Wen.

**Visualization:** Hongyu Chen, Xiaoping Huang, Gang Yuan.

**Formal analysis:** Shaochun Lu.

**Conceptualization:** Zhihong Zhang, Gang Yuan.

**Supervision:** Gang Yuan.

**Validation:** Gang Yuan.

**Writing – original draft:** Hongyu Chen, Xiaoping Huang.

**Writing – review & editing:** Zhihong Zhang, Gang Yuan.
